# Post-COVID-19 Pandemic Sequelae in Liver Diseases

**DOI:** 10.3390/life15030403

**Published:** 2025-03-04

**Authors:** Cristina Stasi

**Affiliations:** 1 Department of Life Science, Health and Health Professions, Link Campus University, 00165 Roma, Italy; cristina.stasi@gmail.com or c.stasi@unilink.it; 2 Epidemiology Unit, Regional Health Agency of Tuscany, 50141 Florence, Italy

**Keywords:** COVID-19, long COVID, liver diseases, metabolic-associated fatty liver disease, metabolic dysfunction-associated steatohepatitis, secondary biliary cholangitis, liver injury, transaminases, fibrosis, viral hepatitis

## Abstract

During the coronavirus disease 2019 (COVID-19) pandemic, several studies highlighted a worse prognosis for patients with alterations in liver function tests, especially those with pre-existing liver diseases. However, further studies are needed to define the long-term impact of the COVID-19 pandemic on liver diseases. Long COVID-19 encompasses a wide range of signs and symptoms, including exacerbations of pre-existing chronic conditions or new onset conditions developed after the COVID-19 acute phase. Therefore, the long-term effects of COVID-19 extensively include hepatic manifestations. The co-expression of angiotensin-converting receptor 2 (ACE2) and transmembrane serine protease 2 (TMPRSS2) has been demonstrated also in enterocytes, cholangiocytes, and hepatocytes. Studies on the post-COVID-19 sequelae have shown the presence of steatosis and necroinflammation in the liver, concomitantly with an alteration of inflammation, cytolysis and cholestasis indices. Some studies also demonstrated an increased risk for hepatobiliary pathologies, including secondary biliary cholangitis and worsening of the severity of metabolic-associated fatty liver disease (MASLD). Based on these premises, this review aims to provide an overview of the pathophysiological mechanisms contributing to COVID-19-related liver and hepatobiliary damage; explore its implications for liver inflammation and fibrosis, with a particular focus on MASLD and metabolic dysfunction-associated steatohepatitis (MASH); and analyze the short- and long-term COVID-19 sequelae. A literature search was conducted using the PubMed database for relevant studies published in English.

## 1. The State-of-the-Art

During the coronavirus disease 2019 (COVID-19) pandemic, several studies indicated that liver injury was a frequent complication of COVID-19 [1]. A meta-analysis conducted by Li et al. [1] evaluated the incidence and risk factors of liver damage in COVID-19 patients, including 49 studies for a total of 23,611 patients, showing a prevalence of hepatic damage of 39.63%. The findings showed that COVID-19-related liver damage was substantially associated with the male gender, middle and elderly age, previous chronic liver disease, inflammatory state, and some drugs. Furthermore, these patients showed longer hospitalizations and more frequently presented with poor prognoses in comparison with patients without liver damage.

In December 2022, studies by the World Health Organization [2] highlighted that 10–20% of severe acute respiratory syndrome coronavirus 2 (SARS-CoV-2)-infected subjects developed symptoms attributable to long COVID. NICE guidance [3] (last updated in 2024) indicates that the definition of “long COVID” is the syndrome characterized by the presence of signs and symptoms that continue or emerge after the COVID-19 acute phase, including both ongoing symptomatic COVID-19 syndrome (4 to 12 weeks after the acute phase) and post-COVID-19 syndrome (12 weeks or more after the acute phase). As important features of long COVID, Ely et al. [4] include the exacerbation of preexisting health conditions or the insurgence of new conditions.

Regarding post-COVID-19 syndrome, de Lima et al. [5] studied changes in serum markers of liver injury for up to 20 months in patients with long-term effects of COVID-19. The most common symptoms in this cohort of 243 patients were fatigue and dyspnea (70%). The most frequent comorbidity was arterial hypertension. The study demonstrated an alteration in serum levels of alanine aminotransferase (ALT), aspartate aminotransferase (AST), lactate dehydrogenase (LDH), and gamma glutamylaminotransferase (GGT) in patients who developed long-term COVID-19, particularly those admitted to hospital during the acute phase, with persistent liver damage 1.5 years after recovery from the COVID-19 acute phase.

SARS-CoV-2 infects host cells by binding angiotensin-converting receptor 2 (ACE2) using a spike glycoprotein (s protein), while transmembrane serine protease 2 (TMPRSS2) ensures its entry into the cell [6,7].

The co-expression of ACE2 and TMPRSS2 was demonstrated in lung cells, enterocytes of the small intestine and colon [6], and cholangiocytes and hepatocytes [7]. Fondevila et al. [8] demonstrated that these genes were more expressed in obese patients with metabolic dysfunction-associated steatohepatitis (MASH) [9], previous non-alcoholic steatohepatitis (NASH). Metabolic-associated fatty liver disease (MASLD), formerly known as non-alcoholic fatty liver disease (NAFLD), encompasses a spectrum of fatty liver diseases from steatotic liver disease (SLD), characterized by ≥5% hepatic steatosis [9], to the MASH, its more severe and progressive manifestation, which affects about 5% of the adult population worldwide [10], as well as fibrosis, cirrhosis, and MASH-related hepatocellular carcinoma (HCC) [11]. The terminology has evolved from NAFLD to MAFLD (Metabolic Associated Fatty Liver Disease) and, most recently, MASLD [11]. This review will utilize the most recent nomenclature, focusing on MASLD and MASH, given that patients with these conditions may be at increased risk of COVID-19 disease progression [8].

Although many studies have investigated liver damage in hospitalized COVID-19 patients during the pandemic, as well as worse outcomes in patients with liver injury [1], there are still gaps in the literature regarding an overview of the most recent findings on the short- and long-term effects of liver and hepatobiliary injury. Understanding existing gaps and exploring recent research on this topic could be critical for the diagnostic and therapeutic management of patients with post-COVID hepatic manifestations.

Based on these premises, this review aims to provide a brief overview of the pathophysiological mechanisms contributing to COVID-19-related liver and hepatobiliary damage. It will explore the implications of COVID-19 on liver inflammation and fibrosis, with a particular focus on MASLD and MASH, analyzing the short- and long-term consequences of COVID-19 in studies published in the last two years. A literature search was conducted using the PubMed database for relevant studies published in English.

## 2. The Burden of Liver Disease in the Post-COVID Era

The COVID-19 pandemic, combined with increased exposure to unhealthy diets and physical inactivity, led to more people becoming obese [12]. Liver diseases frequently rapidly progress in patients with obesity and cardiometabolic comorbidities and pharmacological treatments [11]. In a narrative review, Adenote et al. [13] highlighted that obesity, hypertension, and diabetes, conditions commonly present in MASLD, significantly increase the risk for a severe form of COVID-19. In 2022, a meta-analysis and systematic review [14] reported an overall prevalence of MASLD of 38.77% (95% confidence interval (CI) 32.94% to 44.95%). Danpanichkul et al. [15], analyzing data from the Global Burden of Disease study between 2000 and 2021, found that the incidence of MASH-associated primary liver cancer in young adults was significantly increasing. In particular, the age-standardized death rate and age-standardized disability-adjusted life years were found to decrease in all other etiologies, except for MASH-associated primary liver cancer, in which they remained unchanged. From 2000 to 2021, MASH-related primary liver cancer in young adults represented 6%, which resulted in an increase of incident cases of +1% (since 2000), as well as increased by 6% (+2% since 2000) deaths, and 6% disability-adjusted life year (+2% since 2000) of all primary liver cancer in young adults [15]. In particular, primary liver cancer, in young adults was estimated to be 4300 (95% UI: 3310–5510), with an annual percent change from 2000 to 2021 of 0.26 (95% UI: 0.16–0.35) [15]. Kim et al. [16] in the United States (US) demonstrated that the prevalence of steatotic liver disease (SLD) and its subclasses remained unchanged, while there was a significant increase in advanced fibrosis among subjects with SLD during the COVID-19 era, with alcohol-associated liver disease (ALD) presenting a higher proportion of advanced fibrosis and cirrhosis. Although SARS-CoV-2 infection could lead to immune dysregulation, few reports correlate it to the appearance of de novo immune-mediated liver diseases, such as primary biliary cholangitis (PBC), and the clinical impact of SARS-CoV-2 infection remains unclear [17]. The median prevalence of PBC is around 13.7/100,000 subjects [18]. According to the guidelines of the European Association for the Study of the Liver (EASL), all PBC patients should be evaluated for their risk of evolving towards complications due, in some cases, to the progression of fibrosis to assess the need for additional treatments [19].

Ito et al. [20] highlighted that hepatocellular carcinoma (HCC) due to MASLD is rapidly increasing, and that MASLD-related HCC may develop in non-cirrhotic patients with a decreased survival rate than viral hepatitis-related HCC. At the same time, ALD is a leading cause of death related to HCC, and its prevalence has increased, linked to the impacts of the COVID-19 pandemic [21]. A Canadian study [22] analyzed the impact of COVID-19 from March to December 2020 on HCC surveillance compared with that before (between January 2018 and December 2020) using data from all hepatitis C virus (HCV) cirrhotic patients present in the British Columbia Hepatitis Testers Cohort dataset. They found a major decrease in screening of HCV cirrhotic patients screened for HCC, showing that surveillance returned to pre-pandemic levels in mid-2020, likely due to strategies such as online consultation implemented during the pandemic period.

## 3. Pathophysiology of COVID-19-Associated Liver and Hepatobiliary Damage

**Figure 1:**
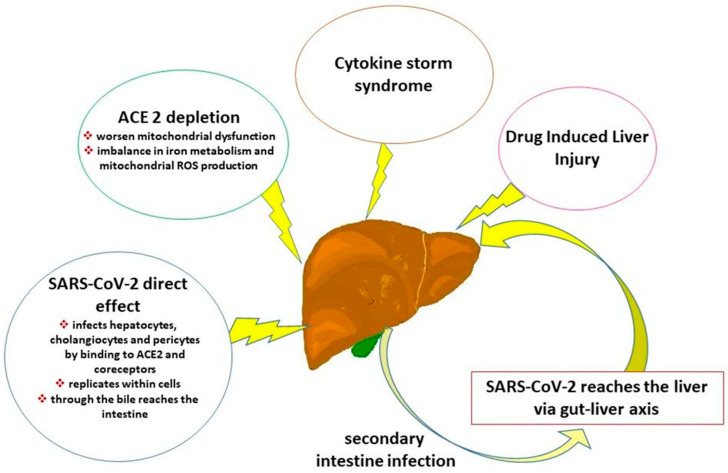
Main pathophysiological mechanisms based on the studies. Legend: SARS-CoV-2 can directly infect the liver by binding to angiotensin-converting receptor 2 (ACE2) receptors on hepatocytes, cholangiocytes, and pericytes, replicate within cells, and reach the intestine through the enterohepatic circulation of bile acids, causing a secondary liver infection. SARS-CoV-2 can instead infect enterocytes, reducing the integrity of the intestinal barrier, entering the portal circulation and reaching the liver (gut–liver axis). ACE2 depletion related to SARS-CoV-2 infection may worsen mitochondrial dysfunction and imbalance in iron metabolism and mitochondrial production of reactive oxygen species (ROS), triggering an apoptotic response. Cytokine storm syndrome proceeds activating intracellular inflammasomes and the release of proinflammatory cytokines, causing prolonged chronic inflammation. Some treatments for COVID-19 patients are hepatotoxic and may lead to drug-induced liver damage.

During the COVID-19 pandemic, alterations of liver enzymes were reported as risk factors for a severe form of COVID-19 in several studies involving hospitalized patients [23,24,25]. Evidence indicates that elevated levels of transaminases are maintained over time [5]. Regarding liver injury, it has been hypothesized [6] that following SARS-CoV-2 infection, an accumulation of proinflammatory cytokines and chemokines [26] may damage alveolar epithelial cells with a consequent spread of the virus from the damaged alveoli to the capillaries and, after damaging the intestinal mucosal epithelium and the vascular barrier, the virus enters the liver via the portal vein. SARS-CoV-2 enters hepatocytes by binding to ACE2 and coreceptors [6,26,27]. After the virus infects hepatocytes and cholangiocytes, SARS-CoV-2 can reach the intestine through the bile, causing a secondary intestine infection [6] (Figure 1). SARS-CoV-2 may infect endothelial cells by attaching to ACE2 receptors, replicating within cells, and releasing into the bloodstream. This facilitates the spread of the virus to other organs, including the liver [27]. Cadamuro et al. [28] demonstrated that in the procoagulant phenotype of COVID-19, SARS-CoV-2 infection primarily targets pericytes, located in the portal wall of the microcirculation.

Regarding biliary and hepatobiliary injury, SARS-CoV-2 can infect and replicate in biliary epithelial cells [29]; since bile is secreted in the intestine, SARS-CoV-2 is also capable of infecting the gastrointestinal tract [6,29] (Figure 1). Leonhardt et al. [29] observed a significant increase in the incidence of secondary sclerosing cholangitis in critically ill patients (SSC-CIP), suggesting that this complication is linked to confirmed risk factors for SSC-CIP, such as hyperfibrinogenemia, rather than to specific SARS-CoV-2-related etiology. Hyperfibrinogenemia may contribute to bile duct hypoxemia and represents an independent risk factor for the SSC-CIP occurrence.

Additionally, another route for SARS-CoV-2 to reach the liver is through the gut-liver axis. Several pieces of evidence have shown that SARS-CoV2 infects enterocytes, alters the intestinal microbiota, and destroys the tight and adherens junctions of the endothelium and intestinal epithelium, which in turn can lead to leaky gut syndrome [30] and then can reach the liver via the portal vein [6].

Cano et al. [31] observed a relationship between MASH and an increased ACE2 expression in liver sinusoidal endothelial cells and hepatocytes, correlating with the liver fat area, inflammation, increased immune reactivity, and fibrogenesis. In turn, the increased ACE2 expression in response to metabolic stress by increased lipid load augments susceptibility to infection.

The progression of liver fibrosis seems to depend on the balance between the classical arm of the renin-angiotensin system (RAS), involving ACE-Angiotensin II-AT1 receptor, and the counter-regulatory arm, involving ACE2-Angiotensin-(1–7)–Mas receptor [32,33]. Based on these different arms, ACE2 plays a protective role in liver diseases against oxidative stress, inflammation, and fibrosis. Consequentially, ACE2 depletion in hepatocytes, resulting from SARS-CoV-2 infection, can worsen mitochondrial dysfunction, inducing increased mitochondrial reactive oxygen species (ROS), fatty acid synthesis, and reduced cholesterol synthesis/efflux [34] (Figure 1). Mercado-Gómez et al. [35] showed in infected primary human and murine hepatocytes that the imbalance in iron metabolism and mitochondrial ROS production could partly result from exacerbated mitochondrial activity, triggering an apoptotic response. A recent study on C57BL/6J [36] mice intranasally infected by SARS-CoV2 demonstrated that hepatocytes can support the life cycle of the virus, contributing to the release of viral progeny which in turn can infect other cells. An increase in ALT was found due to the death of infected cells through a cytopathic effect induced by the virus and, on the other hand, immune-mediated cell death during the elimination of the virus, parallel to a marked infiltration of immune cells, was evident at histopathological analysis in liver sections [36].

Napodano et al. [37] highlight that one of the main mechanisms of the progression of COVID-19 is the “cytokine storm syndrome”, with subsequent activation of intracellular inflammasomes and proinflammatory cytokines release (Figure 1). The IL-6 plays a key role in monocyte chemotaxis and sustained chronic inflammation. Specifically, obesity and MASLD have been associated with the increased production of Kupffer cells with proinflammatory cytokines. Together with the polarization of the macrophage state, this could influence the inflammatory response or immune tolerance to SARS-CoV-2 signals triggered by the gut–liver axis of the host [37].

Adenote et al. [13] summarized data on infectious complications in MASLD patients and suggested that the chronic proinflammatory state associated with MASLD and metabolic comorbidities may partly play a role in the activation of the RAS, which in turn promotes cytokine storm and may cause multi-organ failure. Furthermore, some medications used to treat COVID-19 patients are hepatotoxic and can induce drug-induced liver injury. The alteration of the intestinal microbiota, in parallel with the accumulation of visceral adipose tissue, determines an imbalance between pro- and anti-inflammatory cytokines, causing prolonged inflammation in MASLD and triggering an increased risk of developing various infections, including Clostridioides difficile [13].

## 4. COVID-19 Effect on Liver Inflammation and Fibrosis

Zeuzem et al. [38] studied the changes of soluble mediators of inflammation during and after SARS-CoV-2 infection and acute and chronic HCV infections. They found that cirrhotic patients showed the highest activity of soluble inflammatory mediators during long-term follow-up. The cohort, including patients with long-term COVID symptoms, had elevated levels of interleukin 6 (IL6), tumor necrosis factor-alpha (TNFα), and interferon-gamma (IFNγ) at month 9 (Table 1).

Rodriguez-Espada et al. [39] summarized histopathological findings of SARS-CoV-2 on the liver, reporting that, aside from cases with significant liver damage that progressed to acute liver failure, most studies showed portal or lobular inflammation in approximately 50% of cases. Other data found a wide range of steatosis present, which in the various studies considered varies between 30% and 90%. Although the presence of mild fibrosis was reported, it was unclear whether this may be due to a pre-existing cause of liver damage. Wang et al. [40] demonstrated the direct cytopathic effect of SARS-CoV2 through ultrastructural evidence of hepatocytes that showed a conspicuous swelling of the mitochondria.

A recent Systematic Review by Lebbe et al. [41] examined the occurrence of liver damage in subjects who had COVID-19, reporting 500 cases with long-term post-COVID-19 parenchymal liver damage out of a total of 22 studies considered for a total of 161,594 subjects. In particular, the findings reported several hepatic manifestations COVID-19-related, including acute hepatitis with cholestasis and autoimmune hepatitis, acute liver failure, and liver fibrosis. Among these, steatosis, MASLD and cirrhosis were detected as the most frequent liver-associated complications post-COVID-19 [41]. In turn, Bruzzone et al. [42] demonstrated, by metabolomic analysis, an alteration of indicators of hepatic glutathione synthesis and oxidative stress, indicating that SARS-CoV-2 infection induced liver damage through dyslipidemia and oxidative stress.

Gupta et al. [43] studied the influence of COVID-19 on multi-organ and metabolic function in a severe form of COVID-19 hospitalized patients enrolled in the study within 5–7 months of discharge, compared to a control group. The findings showed that patients had increased insulin resistance, without different fuel oxidation throughout the body rates, probably promoted by a pro-inflammatory state induced by the virus. Interestingly, in patients compared with controls, hepatic volume was found to be 28% larger compared with the controls, concomitantly to an increased fat fraction-adjusted liver T1, indicating inflammation. An Australian study [44] evaluated liver stiffness values following COVID-19 infection with mild symptoms by two-dimensional shear wave elastography technology in the 6 months before the procedure, comparing patients with and without prior COVID-19, excluding participants with pre-existing liver conditions. The study found significant differences in liver stiffness values between the control group and the COVID-19 group, with higher values in the latter, even with stiffness values < 9 kPa ruling out compensated advanced chronic liver disease.

Non-invasive serum markers, both indirect and direct, have been proposed as surrogate markers of liver fibrosis. The indirect serum markers are panels of clinical and biochemical parameters not directly linked to extracellular matrix metabolism [45]. Among them, several studies demonstrated the association between high Fibrosis-4 (FIB-4) scores and worse outcomes in hospitalized COVID-19 patients [46,47,48,49,50,51,52]. Specifically, Lucena Valera et al. [53] evaluated changes in this surrogate marker of fibrosis even after discharge, studying the impact of FIB-4 on the prognosis of COVID-19. They found that the FIB-4 at admission was independently associated with mortality. In addition, transaminases and FIB-4 were significantly reduced six months after the resolution of infection. The changes of FIB-4 occurring after 6 months may be related to necroinflammation, which is also suggested by the parallel decrement of liver transaminases. Kolesova et al. [54] found that, in the acute phase of COVID-19 patients, hyaluronic acid (HA) positively correlated with FIB-4, transaminases, lactate dehydrogenase and ferritin, and negatively with blood oxygen saturation. The longitudinal evaluation of a subgroup of patients demonstrated that inflammatory markers and FIB-4 were higher during the acute COVID-19 phase compared with the post-COVID period (3–6 months). These studies confirm a correlation between FIB-4, hepatocellular injury and systemic inflammation. Hatipoğlu et al. [55] found that hospitalized COVID-19 patients with cirrhosis are at risk of long-term liver disease progression after discharge. However, patients with chronic liver disease without cirrhosis showed no changes in FIB-4 and NAFLD Fibrosis Score (NFS) at 1 year.

The direct serum markers are molecules directly correlated to the extracellular matrix metabolism. Among them, Enhanced Liver Fibrosis (ELF) values strongly diagnosed patients with advanced fibrosis or cirrhosis. ELF includes hyaluronic acid, aminoterminal propeptide of type III collagen, and tissue inhibitor of matrix metalloproteinase 1; therefore, all causes that increase these markers can potentially determine an increase in ELF [56]. Núñez et al. [57] found that patients with parenchymal lung abnormalities and liver function test alteration 3 months after SARS-CoV-2 pneumonia had higher ELF scores.

## 5. Short- and Long-Term Consequences of COVID-19 on Liver Diseases

Based on studies, SARS-CoV-2 infection can induce liver and hepatobiliary damage in both individuals with and without pre-existing liver disease.

This section and Table 1 summarize the short (<6 months) and long-term (>6 months) consequences of COVID-19 on liver and hepatobiliary injury.

Regarding liver injury, Bota et al. [58] analyzed the post-acute consequences of liver damage of SARS-CoV-2 infection in the Romanian elderly population over 2 years compared with the <65 years group, excluding patients with chronic liver disease. They found increased levels of fasting glucose and liver enzymes (ALT and AST) in Long-term COVID patients, particularly those aged ≥65 years during post-discharge. Similarly, cholestasis indices (alkaline phosphatase, GGT, total bilirubin), as well as those of FIB-4, NFS, and aspartate aminotransferase to platelet ratio index (APRI) to assess liver fibrosis risk, were elevated in the long COVID groups, particularly in those aged ≥65 years, suggesting long-term liver monitoring in these patients. Metabolic and liver functions improved over time.

Similarly, as mentioned above, de Lima et al. [5] observed elevated transaminase levels in long-term COVID-19 patients, particularly those hospitalized during the acute phase. 

A Chinese cohort study [59] of 23,838 subjects with physical examination data for three consecutive years showed an increase in the abnormal rate of T-wave patterns after the COVID-19 pandemic, especially for subjects aged 45 years or older affected by chronic diseases such as hypertension, hepatic steatosis, and hyperglycemia.

Among 10 hospitals in Türkiye, İnal et al. [60] conducted a multicenter retrospective study analyzing the laboratory and clinical data of 600 patients with positive anti-rods and rings (RR) antibodies. The findings highlighted an anti-RR prevalence increase in the post-COVID-19 period in comparison with the pre-pandemic era. The pathology most frequently associated with anti-RR positivity was an autoimmune disease (19.83%), of which 28.57% was rheumatoid arthritis and 17.65% was autoimmune liver disease. Moreover, 10.83% of the ring and road pattern-positive patients were diagnosed with viral hepatitis, particularly HCV patients previously treated with peginterferon and ribavirin. Given that the RR pattern is not only related to anti-viral treatment used in HCV patients, the authors hypothesized an involvement of immune dysregulation, suggesting a further investigation into the role of this biomarker for prognostic purposes. A systematic review and meta-analysis conducted by Pan et al. [61] on fifteen cohorts of more than 32 million participants found a 54% long-term greater risk of liver disease, with an absolute magnitude of the COVID-19 effect relatively small.

Regarding hepatobiliary injury, a recent systematic review by Rasheed et al. [62] analyzed cases of SSC, referring to post-COVID-19 cholangiopathy. Liver function tests were elevated in 93.8% of patients. Biliary ductal dilatation was found at ultrasound in 41.6% of patients and thickening of the bile duct wall at MRI with contrast enhancement in 47.7% of patients. Leonhardt et al. [29] observed the appearance of SSC after COVID-19 exclusively in critical COVID-19 patients with invasive ventilation, most likely linked to severe tissue hypoxia and circulatory disorders associated with fibrinogen, hypothesizing a significant increase in patients with SSC-CIP in the post-COVID era.

Lee et al. [63] conducted a large-scale, multinational, population-based registry study, including over 22 million participants aged ≥20 years infected with SARS-CoV-2 between 2020 and 2021, and compared these patients with uninfected controls from South Korea, Japan, and the United Kingdom, evaluating the impact of COVID-19 on the long-term risk of digestive, biliary, and liver diseases and other gastrointestinal disorders, based on International Classification of Diseases, Tenth Revision codes for defining outcomes. They found an increased risk of gastrointestinal, hepatobiliary, and other digestive anomalies in patients with long COVID-19 compared to uninfected control subjects. This risk was more evident in patients with a previous severe form of COVID-19, and increased especially in the first three months. Vaccination decreased the risk for digestive diseases but not liver and biliary diseases [63]. Among the studies that evaluated the evolution of pre-existing liver disease, Hartl et al. [64] studied the prevalence of abnormal liver tests in Austrian-hospitalized patients after the first positive test for SARSCoV-2 (polymerase chain reaction, PCR) with previous chronic liver disease and, subsequently, the trend of hepatocellular parameters, cholestatic liver damage, and clinical outcomes in this cohort of patients. The longitudinal evaluation showed that alkaline phosphatase and GGT increased, while transaminases decreased after reaching a high point during acute SARS-CoV-2 infection. Findings showed laboratory signs of cholestatic liver failure in 23.1% of patients with chronic liver disease, particularly those with MASLD/MASH and COVID-19, while 15.4% of patients developed secondary sclerosing cholangitis, which was more frequent in patients with chronic liver disease who have been SARS-CoV-2 infected in comparison with a matched control group with chronic liver disease and pneumonia not due to COVID-19.

Focusing on metabolic risk factors, a large population study highlighted that chronic conditions, including liver steatosis, in the short-term follow-up (<6 months) were associated with T-wave abnormalities, especially for individuals aged ≥45 [59]. MASLD patients, hospitalized for COVID-19, showed a significant rise in FIB-4 index and NFS with normalization on long-term follow-up [55]. Moreover, the SSC primarily occurred among patients with MASLD/MASH and metabolic risk factors in short-term follow-up [63].

Recent studies conducted in Europe and America indicate that patients with a severe form of COVID-19 appear to have a greater risk of developing post-COVID-19 consequences [5,29,55]. Most cohorts studied have an average age above 40 years [5,38,43,44,53,54,55,63,64]. Patients with pre-existing liver disease have a greater likelihood of experiencing short-term [55,63] consequences.

**Table 1:** Studies on post-COVID short- and long-term laboratory/instrumental evaluation of liver and hepatobiliary injury.

	Authors	Study Design	Country	Number of Patients	Study Population	Time Interval Between COVID-19 and Laboratory/Instrumental Evaluation	Short-Term (<6 Months) COVID Effects	Long-Term (>6 Months) COVID Effects	Risk Factor Associated
Liver	de Lima et al. [5]	cross-sectional, quantitative, descriptive, and analytical observational study	Brazil	243 patients, aged ≥18 years	Long COVID (average age approximately 50 years), without prior chronic liver disease	from 30 to 632 days	GGT, ferritin and total bilirubin in men were above the reference values	Increased ALT and AST levels, especially those hospitalized during acute phase	hospitalization, male, >5 long COVID symptoms were associated with short-term long COVID
	Zeuzem et al. [38]	cross-sectional and longitudinal	Germany	142 SARS-CoV-2 patients (two cohrts): 29 acute HCV, 23 chronic HCV, 31 cirrhotic	SARS-CoV-2 (mean age cohort 1 = 47.6; mean age cohort 2 = 50.2), acute HCV (median age = 45 years), non-cirrhotic Chronic HCV (median age = 56 years), cirrhotic chronic HCV (median age = 56 years),	For acute HCV patients: at baseline, at the end of DAA treatment (week 8), and at 3-month follow-up; For Chronic HCV cirrhotic and non-cirrhotic: baseline, end of treatment (week 8) and at 6 and 24 months after the start of DAA; For SARS-CoV-2 patients: 3, 6, 9, and 14 months	More rapid decline in cytokine and chemokine concentrations after SARS-CoV-2 infection (at month 3) in comparison to all HCV cohorts	Some subjects still had elevated soluble inflammatory mediators levels, e.g., IL6, TNFα, IFNγ (after 6–9 months) in comparison with healthy controls	
	Gupta et al. [43]	Clinical Trial (NCT05060497)	UK	21 patients	21 patients post-COVID-19 (median age = 54 years) and 10 controls	within 5–7 months of discharge	Liver volume 28% greater in patients than controls. Similarly, the measure of liver inflammation		
	Lau et al. [44]	prospective case–control study	Australia	34 post-COVID-19 patients	34 post COVID patients (average age of 41.1 years) without pre-existing liver conditions and 34 controls	<2 months post-infection within 2 to <4 months post-COVID-19 <6 months post-COVID-19	COVID-19 group showed significantly higher liver stiffness values than the control group		
	Lucena Valera et al. [53]	retrospective multicenter study	Spain	575 patients requiring admission	Hospitalized COVID-19 patients (mean age = 68 ± 15 years) between January and June 2020	6-months post-infection	AST, ALT, FIB-4 were significantly reduced at six months after the resolution of infection. 68.4% of patients had FIB-4 < 1.45 after the resolution of infection		patients with higher values of FIB-4 (at baseline or at the time of admission), are at high risk of suffering a poor prognosis COVID-19-associated
	Kolesova et al. [54]	cross-sectional, single-center study	Latvia	124 COVID-19 and post-COVID patients	66 COVID-19 patients, 58 post-COVID (mean age = 42.1 ± 13.4 years), 17 control subjects	3–6 months after the recovery	Increased FIB-4 in 5% of patients of post-COVID, of whom 2% had FIB-4 ≥ 3.25, corresponding to advanced liver fibrosis, even with inflammatory markers in the normal range.		
	Hatipoğlu et al. [55]	Research conducted using Patient Data Registry	USA	52 patients hospitalized for COVID-19 with chronic liver disease	52 patients hospitalized for COVID-19 (mean age = 56.8 ± 13.9 years) and 92 controls with chronic liver disease. 42% cirrhotic COVID-19 patients and 26% cirrhotic in control group. NFS was calculated only in MASLD patients	12.2 months after admission	increased 30-day mortality in cirrhotic COVID-19 patients	Hospitalized COVID-19 patients showed a significant rise in FIB-4 index and NFS (in MASLD) with normalization on follow-up; patients with chronic liver disease without cirrhosis did not reveal changes in FIB-4 and NFS at one year	
	Bota et al. [58]	longitudinal research	Romania	238 participants; 117 Long COVID <65 years; 71 Long COVID ≥65 years; 50 no Long COVID	hospitalized COVID-19 patients without chronic liver diseases	six months after hospitalization	Elderly Long COVID patients showed a strong increment of liver enzymes post-discharge		FIB-4, NFS, and APRI to assess liver fibrosis were significantly higher in patients with Long COVID, particularly in the elderly group
	Liu et al., [59]	comparative cohort study	China	23,838 subjects, 6786 with examinations data during the COVID-19 pandemic	large-scale population cohort	comparison of examination indicators between November 2020 and June 2023	T-wave alterations, particularly in subjects >45 years with chronic diseases such as hypertension, liver steatosis, and hyperglycemia		
Hepatobiliary	Leonhardt et al., [29]	ambidirectional observational study	Germany	25 SSC-CIP patients	adults (median age = 59 years) hospitalized COVID-19 pneumonia confirmed by PCR who developed SSC-CIP, compared with control group without SSC-CIP	1 year after SSC-CIP onset		Without transplantation, only 40.0% of patients with SSC-CIP were alive 1 year after SSC-CIP occurrence	Multivariate analysis confirmed high levels of fibrinogen and LDH as independent risk factors for occurrence of SSC-CIP in ventilated COVID-19 patients
	Lee et al. [63]	multinational population-based cohort study	Korea UK USA	90,399 COVID-19; 386,787 Non-COVID-19	SARS-CoV-2 patients (45.83 ± 13.26 years) and non-infected individuals, as controls	<3, 3–6, and ≥6 months	Increased incidence of digestive and hepatobiliary diseases, and other gastrointestinal abnormalities in SARS-CoV-2 patients during the post-acute phase		-The risk for gastrointestinal and hepatobiliary diseases was pronounced according to the COVID-19 severity and during the initial 3 months -SARS-CoV-2 vaccination reduced the risk of gastrointestinal diseases but not hepatobiliary diseases and other digestive abnormalities
	Hartl et al. [64]	retrospective study	Austria	46 patients without advanced chronic liver disease; 19 patients with advanced chronic liver disease	65 Adult hospitalized COVID-19 patients (67.7 ± 19.6) with chronic liver disease	Median follow-up time = 34.5 (IQR 107.0) days	During follow-up, 47.7% of chronic liver disease patients had severe cholestasis; 15.4% of chronic liver disease patients developed SSC; 1 patient with preexisting primary sclerosing cholangitis showed disease progression; 26.3% of advanced chronic liver disease patients had a decompensation event		COVID-19–associated SSC occurred predominantly in patients with MASLD/MASH and metabolic risk factors.

**Abbreviations:** COVID-19: coronavirus disease 2019; GGT: gamma-glutamyl aminotransferase; ALT: alanine aminotransferase; AST: aspartate aminotransferase; SARS-CoV-2: severe acute respiratory syndrome coronavirus 2; HCV: hepatitis C virus; DAA: direct-acting antiviral agents; IL6: interleukin 6; TNFα: tumor necrosis factor-alpha; IFNγ: interferon gamma; MASLD: metabolic-associated fatty liver disease; NFS: NAFLD Fibrosis Score; APRI: aspartate aminotransferase to platelet ratio index; SSC: secondary sclerosing cholangitis; SSC-CIP: secondary sclerosing cholangitis in critically ill patients; IQR: interquartile range; MASH: metabolic dysfunction-associated steatohepatitis.

## 6. Management Strategies and Future Perspectives

COVID-19 vaccines strongly protect against symptomatic infection and reinfection [65]. Cornberg et al. [66] emphasize that vaccination against various pathogens, including SARS-CoV-2, administered as soon as possible to patients with chronic liver diseases, is a crucial preventive measure, particularly for patients presenting an increment of infection risk and, consequently, mortality, such as those with cirrhosis, hepatobiliary tumors, liver transplant candidates, and immunosuppressed patients after liver transplantation [66]. Several pieces of evidence demonstrate that MASLD patients are more vulnerable to infection [13,35]. This evidence opens up various scenarios that go beyond SAR-CoV-2 infection. After three COVID-19 vaccines, patients with liver disease showed strong antibody and T-cell responses to vaccination and tended to experience a mild form of COVID-19 [67]. However, some of these patients do not respond adequately to vaccination due to compromised immune systems [67,68], including cirrhotic patients [68], liver transplant recipients [67], and also MASLD patients 6 months after their third dose of the inactivated vaccine [69], placing the indication for further assistance in monitoring patients who are more vulnerable to hyporeactivity to SARS-CoV-2 vaccines.

During the COVID-19 pandemic, based on the pathological features and clinical phases of COVID-19, particularly in patients with moderate to severe COVID-19, different classes of drugs were used such as antiviral agents, monoclonal antibodies, inflammation inhibitors/antirheumatic drugs, low molecular weight heparins, plasma, and hyperimmune immunoglobulins [70]. Boettler et al. [71], in the EASL ESCIMID position paper, provided recommendations for patients with liver disease and underlined the importance of considering potential adverse events associated with certain drugs in these patients. This is particularly crucial for patients undergoing immunosuppressive therapies and those with impaired liver function (e.g., in patients with Child-Pugh B/C cirrhosis), who are at a high risk of drug toxicity. An update on the previous position paper [72], which summarizes the evidence of liver disease involvement during COVID-19, provides specific recommendations for patients with chronic liver disease. In MASH patients, a sedentary lifestyle negatively affects metabolic conditions and contributes to disease progression, therefore, changes in lifestyle and nutrition, and weight loss and diabetes management, are recommended [72]. In this context, Mercado-Gómez et al. [35] suggest that metformin could prevent liver dysfunction caused by SARS-CoV-2 in MASLD and type 2 diabetes by improving mitochondrial respiration, reducing Complex I activity through activation of 5′ AMP-activated protein kinase, and a concomitant reduction in angitensin levels (1–7) or jaundice.

Based on the premise that the ketogenic state is protective of tissues and organs, while the hyperinflammatory responses observed in acute COVID-19 can also occur in long COVID-19, an ongoing trial [73] evaluates the effects of nutritional management for patients with long COVID-19. The trial involves planning a low-carbohydrate diet that emphasizes avoiding foods containing sugars and starch while increasing the intake of healthy fats and protein sources. Lau et al. [74] conducted a randomized, double-blind, placebo-controlled study in patients with post-acute COVID-19 syndrome, randomly assigned (1:1) to receive SIM01 (10 billion colony-forming units in sachets twice daily) or placebo orally for 6 months. The findings demonstrated that treatment with SIM01 alleviates multiple symptoms of post-acute COVID-19 syndrome. Given the bidirectional connections between the gut and liver and the established role of probiotics in liver disease, further studies could explore the potential improvement of post-COVID-19 sequelae in liver disease patients.

The precise diagnosis and treatment algorithm for long-term COVID consequences in the liver disease state is still developing, and further studies are needed to define the trajectory of these consequences.

## 7. Conclusions

After the COVID-19 pandemic, there has been an increased incidence of MASH-associated primary liver tumors worldwide and obesity, largely due to lifestyle changes. Long-term follow-up is crucial in cases of persistent liver function alterations in previous COVID-19-infected patients with or without preexisting liver disease. Many studies have demonstrated the presence of necroinflammation, hepatic steatosis, and increased levels of proinflammatory cytokines and inflammation indices in subjects with post-COVID-19 sequelae (Table 1), suggesting careful monitoring of patients with altered levels of hepatic cytolysis and cholestasis indices.

Research conducted across different cohorts of patients indicates that necroinflammatory activity [5,58] and inflammatory mediators [38] persist even after 6 months from infection. The hepatobiliary diseases linked to COVID-19 predominantly occurred in patients within 6 months from discarge [63,64], especially among patients with MASLD/MASH and metabolic risk factors [64]. Therefore, long-term surveillance studies in these patients are needed to evaluate whether fibrosis progresses as a long-term effect of triggered necroinflammation. This is particularly relevant in the context of MASLD, where COVID-19 may lead to additional fat accumulation in the liver, secondary to increased serum levels of triglycerides during infection [75] and lipid accumulation in virus-induced compartments [76].

## Data Availability

All the data reviewed in this manuscript are available online, particularly in PubMed.
